# Keeping Older Adults at Home: Prediction Modeling for 30-Day Outcomes After Sepsis Discharge

**DOI:** 10.1093/geroni/igaf122.3800

**Published:** 2025-12-31

**Authors:** Sang Bin You, Jiyoun Song, Miriam Ryvicker, Yolanda Barron, Kathryn Bowles

**Affiliations:** University of Pennsylvania, Philadelphia, Pennsylvania, United States; University of Pennsylvania School of Nursing, Philadelphia, Pennsylvania, United States; VNS Health, New York, New York, United States; VNS Health, New York, New York, United States; University of Pennsylvania School of Nursing, Philadelphia, Pennsylvania, United States

## Abstract

Many older adults prefer to remain in their homes and communities, yet sepsis survivors receiving home healthcare (HHC) often experience readmissions or transition to other facilities. This study aims to identify modifiable risk factors that can alert clinicians and trigger interventions to keep older sepsis survivors at home. We analyzed Medicare fee-for-service beneficiaries (2021-2022) hospitalized for sepsis and discharged with HHC using claims and home care assessment data. The outcome, days-not-at-home, was defined as binary indicator of whether patients spent time in hospitals, skilled nursing, or long-term facilities within 30 days. Logistic regression, random forest, neural network, and XGBoost models were developed, tuned with cross-validated grid search, and evaluated using 10-fold cross-validation. Performance was assessed using the area under the receiver operating characteristic curve (AUC). The cohort included 19,019 older adults [mean age 78.5 years, standard deviation (SD)=8.1] with an average of 5.5 chronic conditions (SD = 2.0), mean hospital stay of 8.1 days (SD = 7.0), and nearly all (96.9%) used ≥5 medications. Within 30 days, 25.5% experienced days-not-at-home. The neural network performed best (AUC=0.6052), followed by logistic regression (AUC=0.6044), XGBoost (AUC=0.6037), and random forest (AUC=0.5955). Top predictors included receiving enteral nutrition therapy, having an ostomy, ≥2 hospitalizations in the past 6 months, positive or missing depression screening, and having pressure ulcer(s). Although overall accuracy was modest, results suggest that addressing depression, enhancing nutrition support and providing close scrutiny of wounds may reduce risk and prevent institutional care among sepsis survivors. Findings may guide risk stratification and tailored interventions to reduce preventable utilization.

